# What Do Anesthesiologists Know about* p* Values, Confidence Intervals, and Correlations: A Pilot Survey

**DOI:** 10.1155/2017/4201289

**Published:** 2017-10-12

**Authors:** Patrick Schober, Sebastiaan M. Bossers, Phi-Vu Dong, Christa Boer, Lothar A. Schwarte

**Affiliations:** ^1^Department of Anesthesiology, VU University Medical Center, De Boelelaan 1117, 1081 HV Amsterdam, Netherlands; ^2^Department of Anesthesiology, Catharina Hospital, Michelangelolaan 2, 5623 EJ Eindhoven, Netherlands

## Abstract

**Background:**

Statistical methods form the basis for clinical decision-making in evidence-based anesthesia. Data on the knowledge of anesthesiologists about statistics are lacking. This pilot study aims to provide a first impression of the anesthesiologists' understanding of commonly used concepts in statistics.

**Methods:**

A cross-sectional pilot survey was performed at a major international anesthesia conference. The questionnaire consisted of three basic multiple-choice questions on the topics “*p* value,” “confidence interval,” and “correlation.” Results of the questions are reported as percentage of correct answers (95% confidence interval).

**Results:**

65 questionnaires were analyzed. Forty participants were male, and mean age was 40 (standard deviation: 10) years. The question addressing the* p* value was correctly answered by 15% (95% CI: 8 to 27%) of respondents. The question concerning the 95% confidence interval was answered correctly by 28% (95% CI: 18 to 40%) of participants. For the question about correlation, a correct answer was given by 52% (95% CI: 40 to 64%). None of the participants answered all questions correctly, and 19 participants provided a wrong answer to all questions.

**Conclusions:**

Anesthesiologists seem to demonstrate a poor understanding of statistical key concepts. Further studies are needed to address statistical knowledge gaps among anesthesiologists more comprehensively.

## 1. Introduction

Evidence-based medicine heavily relies on the results of scientific research. Statistical methods are a cornerstone of the analysis and interpretation of research data and form the basis for clinical decision-making in anesthesia. In order to critically appraise the results and conclusions of clinical and experimental studies, clinicians require an understanding of statistical key concepts [1]. In fact, the* American Board of Anesthesiology* and the* European Society of Anaesthesiology* have recognized the relevance of basic statistical knowledge for clinical anesthesiologists and address this topic during their respective examination procedures [2, 3].

Despite the importance of statistics in anesthesia practice, data on the knowledge of anesthesiologists about statistics are lacking. Identification of knowledge gaps may have practical implications for training curricula. As a first step to assess whether lack of statistical knowledge among anesthesiologists might be a concern that may require further action, we performed a pilot survey to test the understanding of anesthesiologists of three of the most basic and commonly used concepts in inferential statistics:* p* values, confidence intervals, and correlations.

## 2. Methods

We performed a cross-sectional pilot survey at the Annual Meeting of the European Society of Anaesthesiology (ESA) 2015 in Berlin, Germany. The ESA congress is the major anesthesia conference in Europe and was attended by 6637 participants from 107 countries from all over the world. Since it was not feasible to draw a genuine random sample from all delegates, we used a convenience sampling technique and approached individuals in the poster area, in the industry exhibition area, and passing by a corridor that connects two parts of the congress building. Individuals were invited to participate on a voluntary basis, and all answers were collected anonymously. Beside a set of demographic questions about the respondent, the questionnaire consisted of three multiple-choice questions, one each on the topics of “*p* value,” “confidence interval,” and “correlation” (see Original Questionnaire Used in the Survey). For each question, four answers were provided, of which only one was correct. The participants were given as much time as they needed to read, understand, and answer the questions.

### 2.1. Statistics

Sample size estimations and analysis of results were performed with Stata 13.1 (StataCorp, College Station, TX, USA). For this pilot survey, we determined that a margin of error of 10–15% on a 95% confidence level is acceptable for an initial estimate of the magnitude of the percentage correct answers to any given question. A sample size of 61 participants was sufficient for a margin of error of 12.5%. Descriptive statistics are used to characterize the sample of respondents, and results of the multiple-choice questions are reported as percentage of correct answers (95% confidence interval).

## 3. Results

Of 66 completed questionnaires, one was excluded because the respondent had only filled in the demographic part but did not provide an answer to any of the statistical questions. The remaining 65 respondents provided complete answers to the statistical questions.

Forty of the participants (62%) were male, and mean age of the participants who reported their age (*n* = 57) was 40 (standard deviation 10) years. Six participants did not disclose their country of origin, and the other 59 participants came from 23 different countries from 5 continents. Forty-four of the participants were anesthesiologists and 17 anesthesiologists in training, 2 participants did not report their function, and 2 had other anesthesia related functions (one anesthesia nurse, one professor of intensive care medicine). Eleven of the participants (17%) report that they hold academic degrees compatible with advanced statistical training (Ph.D., Associate Professor or Professor), and 43 (66%) of participants have themselves authored or coauthored research papers that report statistics.

The first question about the* p* value was correctly answered by 15% (95% CI: 8 to 27%) of respondents. The second question, concerning the 95% confidence interval, was answered correctly by 28% (95% CI: 18 to 40%) of participants. For the third question about correlation, a correct answer was given by 52% (95% CI: 40 to 64%). None of the participants answered all questions correctly, and 19 participants (29%) provided a wrong answer to all questions.  Table 1 shows the distribution of answers per question and also reports results excluding the four participants who were not anesthesiologists.

## 4. Discussion

In this pilot survey, we tested the knowledge of anesthesiologists about* p* values, confidence intervals, and correlations. The rather limited percentage of correct answers that we observed suggests a poor understanding of these statistical key concepts among anesthesiologists.

*p* values, confidence intervals, and correlations are ubiquitously reported in medical literature and play a key role in statistical estimation and significance testing. Despite the importance of statistics for clinical decision-making in anesthesia and other fields of evidence-based medicine, previous data on the knowledge of physicians about statistics are scarce. Almost 30 years ago, Wulff et al. observed that most physicians lacked the statistical knowledge necessary to draw correct conclusions from medical research [4]. Some 20 years later, Novack et al. and Windish et al. still observed a similarly limited level of statistical understanding [5, 6]. In recent years, attempts have been made by the medical community to improve the knowledge of statistics among clinicians, for example, by emphasizing statistical topics in training and examination curricula, or by regularly publishing dedicated statistical review articles in leading medical journals (e.g.,* “Jama Guide to Statistics and Methods”* or “*Statistics Notes*” in the BMJ). It is therefore unclear whether the previously shown poor knowledge of statistics is still a concern at present; moreover, data on the knowledge of anesthesiologists were completely lacking. We therefore performed a pilot survey at a major anesthesia conference. Although* p* values are reported in virtually all original articles in anesthesia literature, only a minority of the participants knew that the* p* value describes the probability to observe a result at least as extreme as the one that was observed, under the assumption that the null hypotheses were actually true. Less than one-third knew that a 95% confidence interval contains the true population parameter in approximately 95% of the cases if samples are repeated over and over again. Only about half of the participants correctly answered that correlation describes the strength of a relationship between two variables. For a detailed overview about what* p* values, confidence intervals, and correlations are (and importantly, what they are not), we refer the interested reader to published tutorials [7–9].

The poor knowledge that we observed is especially alarming as anesthesiologists with an above average interest in scientific research may likely have been overrepresented in our sample of delegates of an international conference. In fact, a majority of the participants of our survey have themselves authored or coauthored scientific manuscripts, and the rather poor knowledge of statistics casts doubts on whether these participants had been able to use statistical methods appropriately in their own research projects. In this context, inappropriate use of statistical methods has frequently been observed in published manuscripts, even in high impact journals [10–12]. This underlines that anesthesiologists cannot necessarily rely on editors and reviewers to filter out erroneous conclusions based on inappropriate statistics but should themselves be able to judge whether the conclusions are actually supported by the data. Our data suggest that anesthesiologists may often lack the knowledge to do so and that more rigorous training of statistical topics might be useful.

### 4.1. Limitations

There is no validated questionnaire to test statistical knowledge among anesthesiologists. Therefore, our questionnaire was designed from scratch. Herein, we did not aim to comprehensively address knowledge of intricate statistical topics, rather, we aimed to test very basically whether anesthesiologists could identify the correct definition of a* p* value, whether participants knew what a 95% confidence interval is, and whether they understood what correlation describes. Nonetheless, this pilot questionnaire can be further improved for future use. While the answers that we considered correct are definitely the best answer to a given question (and the other answers are definitely wrong), they still may leave some room for discussion. For example, answer B to question 1 correctly states what a* p* value ideally* should* describe. However, in reality, data are often biased and assumptions of statistical tests are usually not met in real-life data analysis. Hence, the* p* value that is reported as a result of a statistical test is not exactly what it claims to describe.

Statistical knowledge may vary among anesthesiologists/trainees from different geographic, academic, and demographic backgrounds, depending on their statistical education during pregraduate and postgraduate studies. To get an impression of the statistical knowledge of the overall group of anesthesiologists, we performed the survey at a major international anesthesia conference with a diverse group of delegates from all over the world. Nonetheless, participants of a conference may also not be representative for anesthesiologists as a whole. Given the limitation that the conference delegates are not a random sample of “the anesthesiologists,” we did not attempt to, and it was not feasible to, draw a genuine random sample from all participants. Rather, a convenience sample of individuals with different geographic and demographic background was used as surrogate for the group of anesthesiologists as a whole. We did not explicitly ask questions about the statistical education of the survey participants in the demographic part of the questionnaire.

Sixty-five individuals participated in our survey, including 61 individuals who reported to be anesthesiologists or anesthesiologists in training. This sample size had been calculated as sufficient to obtain a margin of error of 12.5% on a 95% confidence level. For this pilot survey, it was not our aim to provide a very accurate estimate of the actual knowledge among anesthesiologists. Rather, we aimed to determine as a proof-of-principle whether lack of knowledge on statistics among anesthesiologists might be a concern. For all practical purposes, it is irrelevant whether the actual percentage of anesthesiologists who know the correct answer to any question is 10, 20, or even 40%. While a larger sample size may have resulted in a better estimate, the key message that a substantial proportion of anesthesiologists demonstrate poor knowledge on statistical key concepts remains unaffected by the given sample size. Further studies are needed to address more comprehensively which knowledge gaps need to be addressed and how knowledge on statistics can be best improved.

## 5. Conclusion

Although statistics play a key role in clinical decision-making in anesthesia, anesthesiologists visiting a major European conference demonstrated a poor understanding of statistical key concepts. More emphasis on training of statistical topics may contribute to the improved understanding of statistical concepts. 

*Original Questionnaire Used in the Survey: The Anaesthetist's Questionnaire on Statistics*. The aim of this* anonymous* questionnaire is to test knowledge of commonly used statistical concepts.* Please fill in only one answer per question*.

  Gender:
    □ Male
    □ Female
  Age: —
  Country: —
  I am:
    □ Anaesthesia Resident
    □ Anaesthesiologist
    □ other —
  How many research papers that report statistics have you approximately published in your career? — (Irrespective of first/senior or co-authorship).
  Highest academic title:
    □ none
    □ Master or MD
    □ PhD
    □ Assoc. Prof.
    □ Prof.
(1) What is a “*p*-value” that is reported in the context of statistical hypothesis tests?
   (A) The probability that the null-hypothesis is true given the observed result.
   (B) The probability of observing a result as extreme or more extreme as the observed result if the null-hypothesis were true.
  (C) The probability that the observed result is due to random chance if the alternative hypothesis were true.
  (D) The probability of making a type I error when rejecting the null hypothesis.
(2) Which of the following statements about the 95% Confidence Interval (CI) is true?
  - The 95% CI approximately contains 95% of the sample data.
  - The probability is 0.95 that the 95% CI contains the true population parameter (e.g., population mean).
  - In normally distributed data, the 95% CI is the sample standard deviation multiplied by 1.96.
  - If samples were taken many times and the 95% CI was computed for each sample, about 95% of the computed confidence intervals would contain the population parameter (e.g., population mean).
(3) Which of the following statement about correlation is true?
  - Correlation describes the strength of agreement between two variables.
  - Correlation describes a linear function with which one variable can be predicted from another variable.
  - Correlation describes the extent of changes in the value of one variable that is caused by a change in another variable.
  - Correlation describes the strength of a relationship between two variables.

Thank you for completing this questionnaire!

The results will be processed anonymously.

## Figures and Tables

**Table 1:** Survey results.

Question	Answer	All participants		Only anesthesiologists	
		(*n* = 65)		(*n* = 61)	
		*N*	Percentage (95% CI)	*N*	Percentage (95% CI)
1	A	19	29 (19 to 42)	18	30 (19 to 42)
	*B*	*10*	*15 (8 to 27)*	*9*	*15 (8 to 26)*
	C	22	34 (23 to 46)	21	34 (23 to 47)
	D	14	22 (13 to 34)	13	21 (13 to 34)

2	A	9	14 (7 to 25)	7	11 (5 to 23)
	B	23	35 (25 to 48)	22	36 (25 to 49)
	C	15	23 (14 to 35)	15	25 (15 to 37)
	*D*	*18*	*28 (18 to 40)*	*17*	*28 (18 to 41)*

3	A	14	21 (13 to 34)	14	23 (14 to 36)
	B	8	12 (6 to 23)	7	11 (5 to 23)
	C	9	14 (7 to 25)	8	13 (7 to 24)
	*D*	*34*	*52 (40 to 64)*	*32*	*52 (40 to 65)*

Number (*N*) and percentages of individuals who chose a respective answer to the statistical questions in the survey. Results are reported for all 65 participants of the survey, as well as for the 61 participants who reported that they were anesthesiologists or anesthesiologists in training. The correct answer for each question is displayed in *italic letters*. For the questions and answers, see Original Questionnaire Used in the Survey. CI: confidence interval.

## List of Abbreviations

CI: Confidence interval
ESA: European Society of Anaesthesiology.

## Additional Points

*Availability of Data and Material*. The data used in this study are available from the corresponding author on reasonable request.
